# Association of rotating shift work with incident irritable bowel syndrome: a large population-based prospective cohort study

**DOI:** 10.3389/fpubh.2025.1541122

**Published:** 2025-03-26

**Authors:** Yang Zhong, Hao Bai, Yuan Zhang, Xiaorong Yang, Tongchao Zhang, Xinjie Liu, Zhen Li, Hao Chen, Ming Lu

**Affiliations:** ^1^Department of Epidemiology and Health Statistics, School of Public Health, Cheeloo College of Medicine, Shandong University, Jinan, Shandong, China; ^2^Clinical Epidemiology Unit, Clinical Research Center of Shandong University, Qilu Hospital of Shandong University, Jinan, Shandong, China

**Keywords:** irritable bowel syndrome, shift work, anxiety/depression, sleep quality, prospective cohort study

## Abstract

**Objectives:**

Limited epidemiological study has examined the association between rotating shift work and risk of irritable bowel syndrome (IBS). This study aimed to investigate the association between shift work and risk of IBS and explore the potential mediating factors for the association.

**Methods:**

A total of 268,290 participants from the UK Biobank were included. Cox proportional hazards model was used to examine the associations between shift work and the incidence of IBS. The mediation analyses were performed to investigate the mediating effects.

**Results:**

Participants engaged in always/usually shift work showed a significantly increased risk of developing IBS (HR: 1.12, 95% CI: 1.03–1.23). Joint analysis indicated that, participants with both always/usually shift work and inadequate sleep duration had a 54% increased risk of IBS (HR: 1.54, 95% CI: 1.35–1.82) compared to those with adequate sleep duration and never/rarely shift work; while participants with both always/usually shift work and insomnia-always had a 65% increased risk of IBS (HR: 1.65, 95% CI: 1.43–1.90) compared to those with never/rarely shift work and never/sometimes insomnia. Mediation analysis revealed that sleep quality and anxiety/depression partially mediated the relationship between shift work and IBS incidence, contributing 16.1% (6.8–25.4%) and 3.6% (0.4–6.8%) of the mediation effect, respectively.

**Conclusion:**

This study found that participants with always/usually shiftwork status had significantly increased risk of IBS, and this association may partially be mediated by anxiety/depression and sleep quality. Moreover, inadequate sleep duration and usually insomnia may intensify the effect of rotating shift work on the risk of incident IBS.

## Introduction

Irritable bowel syndrome (IBS) is a functional gastrointestinal disorder characterized by abdominal pain associated with bowel movements or changes in bowel habits (1). It is estimated to affect about 10% of the world’s population and causes substantial annual direct and indirect expenditures related to disease management in countries (2). The etiology of IBS is multifactorial, with factors such as stress, gut-brain axis dysfunction, and altered gut microbiota playing significant roles in its development and exacerbation (3–5). Current clinical treatments for IBS primarily focus on alleviating bothersome symptoms such as abdominal pain and irregular bowel habits, while these treatments are either of uncertain efficacy or are associated with adverse events (6). Patients with IBS frequently report comorbid psychiatric disorders, increased suicidal ideation due to their symptoms, and a reduced quality of life (7). Therefore, it is important to identify primary prevention strategies for IBS to reduce the overall disease burden and improve the quality of life of patients.

Shift work, characterized by extended working hours that deviate from the typical schedule and disrupt circadian rhythms, has become increasingly prevalent with socioeconomic advancement (3). This is partly because some occupations have to keep people on the job 24 h a day (such as nurses), and partly because enterprises have to improve production efficiency (8). Rotating shift forms include morning shift/night shift/rotating shift, periodic shift, and irregular shift. Over the past few decades, increasing attention has pointed to the possible negative health effects of shift work. Previous study has shown that the disruption of circadian rhythm is closely related to multiple gastrointestinal diseases (9). The gut-brain-microbiota axis plays a crucial role in the pathophysiology of IBS and the development of conditions linked to shift work and sleep disturbances (10, 11). Shift work, particularly in rotating and night shifts, disrupts the body’s circadian rhythm and can significantly impact gut microbiota composition, leading to dysbiosis (12). This disruption influences the bidirectional communication between the gut and brain, aggravating IBS symptoms (13). However, there is limited evidence regarding the association between shift work and risk of IBS. Most existing studies on the association between IBS and rotating shift work have been conducted among specific occupational groups, limiting their generalizability (14).

To date, emerging evidence has implicated that the co-occurrence of common mental disorders (e.g., anxiety and depression) in IBS patients is prevalent (15), with these disorders overlapping with IBS symptoms and potentially resulting from a combination of genetic, physiological, and psychosocial risk factors (16). Shift work was associated with an increased risk of adverse anxiety/depression outcomes in general, and depressive symptoms in particular (17). In addition, recent researches have demonstrated significant associations between poor sleep quality and the incidence of IBS (16, 18). A systematic review indicates that compared with healthy controls, the prevalence of sleep disorders in IBS patients is higher (19), which may be related to the pathogenesis of IBS. The poor sleep quality is commonly reported among rotating shift workers (20). Several reports have been made that rotating shift workers experience relatively more stress compared to day workers, resulting in more frequent insomnia and experience of poor sleep quality (21, 22). In short, rotating shift work can affect anxiety/depression and sleep quality, which are independently associated with the occurrence of IBS. However, the mediating roles of anxiety/depression and sleep quality in the relationship between shift work and IBS onset remains unclear. In addition, the joint association of shift work and sleep quality (including insomnia status and sleep duration) on IBS development is also unknown.

In this nationally representative survey of adults from UK biobank, we aimed to investigate the association between rotating shift work and risk of IBS and evaluate whether this association is mediated by anxiety/depression and sleep quality. In addition, we assessed the joint association of rotating shift work and sleep quality with risk of IBS.

## Materials and methods

### Study population

The study involved over 500,000 participants from the UK Biobank, a large-scale prospective cohort. Participants were recruited from 22 assessment centers across England, Wales, and Scotland. At these centers, participants completed a touchscreen questionnaire, underwent a face-to-face interview, and underwent a series of physical measurements (23).

**Figure 1:**
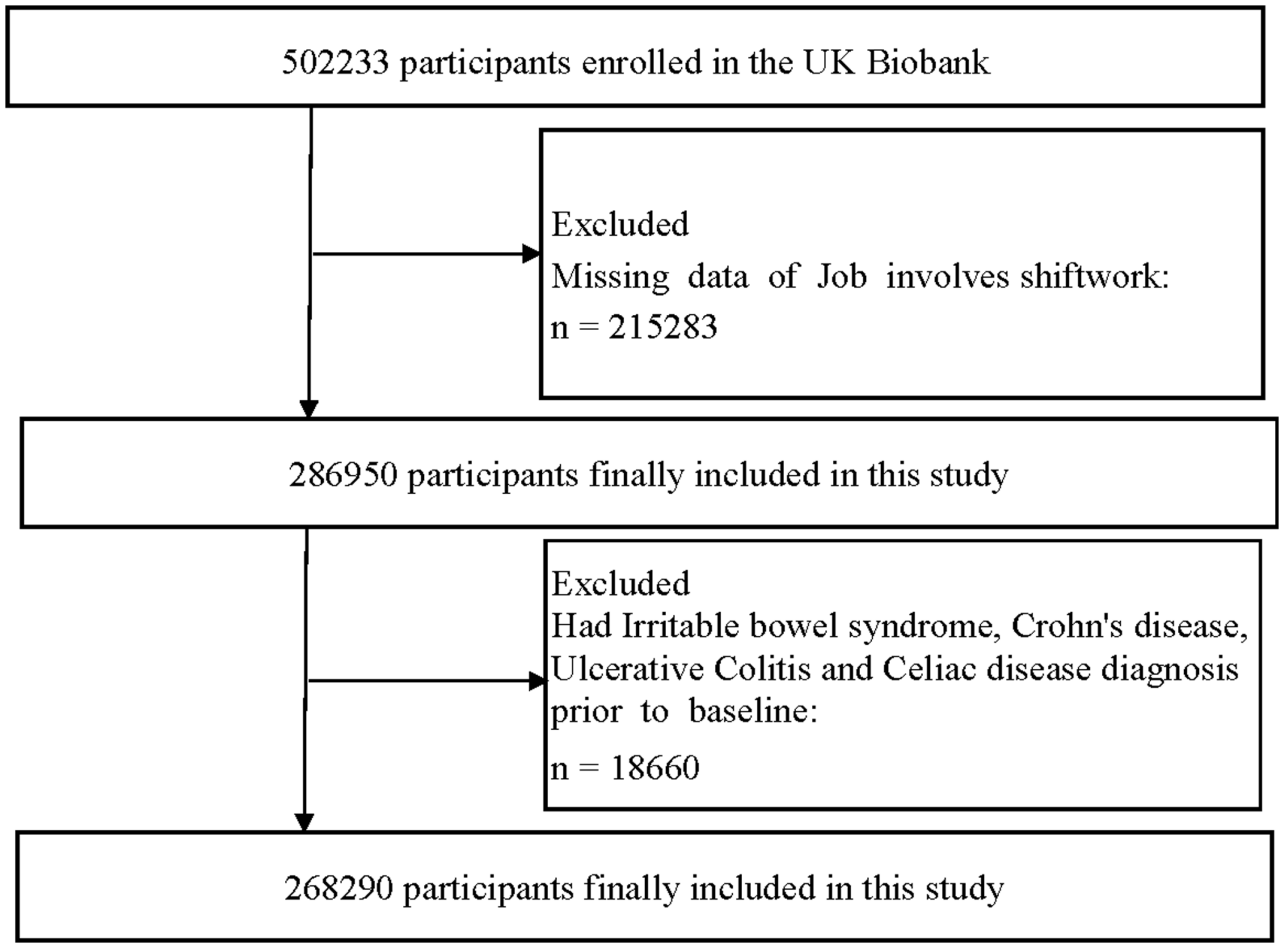
Study overview.

We excluded participants with a diagnosis of IBS and those with missing data of job involves shiftwork at baseline. Participants with the most common benign digestive system diseases with symptoms similar to IBS were also excluded, including patients with Crohn’s disease, ulcerative colitis and celiac disease (Supplementary Table S1). Finally, 268,290 participants were included in this study, as shown in Figure 1.

### Assessment of main exposures

Only participants who were employed at the time of recruitment were considered in shift work. UK Biobank defined shift work as “a schedule falling outside of 9 am to 5 pm.” By definition, participants were classified as “never/rarely shift workers” or “shift workers” based on their current shift work status. Among shift workers, participants were further categorized as “always/usually shift workers” and “sometimes shift workers.”

An index score for the sleep quality was created including 5 aspects of sleep behaviors (24) and in this paper, daytime dozing is used as a substitute for daytime sleepiness. Healthy sleep factors were defined as morning chronotype (“definitely a morning person” or “more a morning than evening person”); adequate sleep duration (adequate); not usually insomnia (never/rarely); no snoring and no daytime dozing (never/rarely or sometimes). Each sleep factor was coded 1 if meeting the healthy criterion and 0 if not. All component scores were summed to obtain a healthy sleep score ranging from 0 to 5, with higher scores indicating a better sleep quality (Supplementary Table S2).

### Ascertainment of outcome

The primary outcome of the study was the incidence of IBS. The diagnosis of IBS was determined using self-report or linkage to death register and/or primary care and/or hospital admission data, based on International Classification of Disease-10 (ICD-10) codes of K58 (Supplementary Table S1).

### Covariates

To reduce the effect of potential confounding, sociodemographic characteristics, lifestyle factors, comorbidities, and markers of inflammation were considered to be covariates based on previous literature (16, 25).

The sociodemographic factors include: age (≤ 50 or > 50 years), sex (female or male), ethnicity (white or not white), Townsend Deprivation Index (< median (−2.09) or ≥ median), and BMI (< 25 or ≥ 25 kg/m^2^). Lifestyle factors variables include smoking status (never or previous), alcohol drinking(never or previous), tea intake (continuous variable), healthy diet score (26) (poor or ideal), optimal physical activity (yes or no: defined as meeting the 2017 UK Physical activity guidelines of 150 min of moderate activity per week or 75 min of vigorous activity), sleep duration (categorized as <6 and ≥ 6 h/day), chronotype preference (definitely a “morning” person, more a “morning” than “evening” person, more an “evening” than a “morning” person, and definitely an “evening” person), daytime dozing (all of the time/often, never/rarely, and sometimes), Snoring (yes or no), sleeplessness insomnia (never, sometimes and usually), anxiety/depression (normal or abnormal) (27). Comorbidities variables include: diabetes diagnosed by doctor (yes or no), high blood pressure diagnosed by doctor (yes or no); markers of inflammation variables include: SII, PLR, NLR, LMR (all categorized as high or low). Calculations were as follows: SII = (neutrophils * platelets)/lymphocytes, NLR = neutrophils/lymphocytes, PLR = platelets/lymphocytes, and LMR = lymphocytes/monocytes (25).

### Statistical analysis

For baseline characteristics, continuous variables were presented as means and standard deviations, and categorical variables were summarized as number and percentages. Univariate comparisons between incident IBS cases and non-IBS were conducted using Student’s t-test or χ^2^ test, respectively. The UK Biobank data were available up to 31 October 2022, which was considered the end of the study period. Person-years of follow-up were calculated from the baseline assessment until the occurrence of IBS, death, loss to follow-up, or the end of the study, whichever came first.

In the primary analysis, a time-to-event analysis of IBS was performed using a Cox proportional hazards model. After collinearity diagnosis, it can be concluded that there is no significant multicollinearity issue in the model. Then, we constructed three models with different covariates to estimate the hazard ratio (HR) and its 95% confidence interval (CI). Crude model was adjusted for nothing; Model 1 was adjusted for age, Townsend Deprivation Index, sex, ethnicity; Model 2 was further adjusted for optimal physical activity, BMI, smoking status, tea intake, alcohol drinking, healthy diet score, chronotype, sleep duration, sleeplessness insomnia, snoring, daytime dozing, high blood pressure diagnosed by doctor, diabetes diagnosed by doctor, anxiety/depression, LMR, PLR, NLR, SII based on Model 1.

As additional exploratory analyses, possible modifications were assessed for variables including age, sex, ethnicity, Townsend deprivation index, BMI, smoking status, alcohol drinking, diabetes diagnosed by doctor, high blood pressure diagnosed by doctor, healthy diet score, optimal physical activity, sleep duration, chronotype preference, sleeplessness insomnia, snoring, daytime dozing, anxiety/depression, LMR, PLR, NLR, SII. Potential modifying effects were assessed by modeling the cross-product term of the stratifying variable with shiftwork variable. The joint association between sleep quality and shiftwork with the risk of IBS were examined using Cox proportional hazard models, which was performed after full adjustment with the combination of never/sometimes insomnia-never/rarely shiftwork or adequate sleep duration-never/rarely shiftwork as the reference. Furthermore, mediation analysis was performed to investigate the mediating effect of anxiety/depression and sleep quality on the shiftwork-IBS association.

Multiple imputation was used for missing data to obtain the final dataset. All *p* values were reported as two-sided tests with significance defined as *p* < 0.05. Statistical analyses were performed in the R software (Version 4.3.2, R Core Team).^1^

## Results

### Baseline characteristics

Table 1 presents the baseline characteristics of the study participants who being in paid employment or self-employment at baseline. Among the 268,290 participants (mean age 52.77 years, 50.9% female), 26,609 (9.92%) participants had always/usually shiftwork behaviors, 221,709 (82.64%) had never/rarely shiftwork behavior, 19,972 (7.44%) participants had sometimes shiftwork behaviors.

**Table 1:** Baseline characteristics.

Variables	All participants		*p*-value
	Non-IBS	Cases of IBS	
	(*N* = 262,931)	(*N* = 5,359)	
Sex			
Female	132,904 (50.5)	3,664 (68.4)	< 0.001
Male	130,027 (49.5)	1,695 (31.6)	
Townsend deprivation index			
Mean ± SD	−1.33 ± 3.01	−1.22 ± 3.03	0.011
Age			
Mean ± SD	52.8 ± 7.10	52.4 ± 6.95	< 0.001
Ethnic			
Not White	16,051 (6.1)	293 (5.5)	0.057
White	246,880 (93.9)	5,066 (94.5)	
BMI			
Mean ± SD	27.3 ± 4.70	27.4 ± 4.97	0.102
Optimal physical exercises			
No	125,286 (47.6)	2,629 (49.1)	0.043
Yes	137,645 (52.4)	2,730 (50.9)	
Job involves shiftwork			
Never/rarely	217,328 (82.7)	4,381 (81.8)	0.033
Sometimes	19,582 (7.4)	390 (7.3)	
Always/usually	26,021 (9.9)	588 (11.0)	
Smoking status			
Current	27,591 (10.5)	562 (10.5)	1.000
Never/previous	235,340 (89.5)	4,797 (89.5)	
Tea intake			
Mean ± SD	3.41 ± 2.90	3.44 ± 3.07	0.441
Alcohol status			
Current	241,776 (92.0)	4,918 (91.8)	0.643
Never/previous	21,155 (8.0)	441 (8.2)	
Healthy diet score			
Mean ± SD	6.91 ± 1.49	6.93 ± 1.49	0.487
Sleep duration			
Mean ± SD	7.05 ± 0.970	6.96 ± 1.09	< 0.001
Chronotype			
Definitely a ‘morning’ person	68,852 (26.2)	1,372 (25.6)	0.013
Definitely an ‘evening’ person	24,097 (9.2)	550 (10.3)	
More a ‘morning’ than ‘evening’ person	93,254 (35.5)	1834 (34.2)	
More an ‘evening’ than a ‘morning’ person	76,728 (29.2)	1,603 (29.9)	
Sleeplessness insomnia			
Never/rarely	74,393 (28.3)	1,025 (19.1)	< 0.001
Sometimes	125,836 (47.9)	2,503 (46.7)	
Usually	62,702 (23.8)	1831 (34.2)	
Snoring			
No	161,151 (61.3)	3,423 (63.9)	< 0.001
Yes	101,780 (38.7)	1936 (36.1)	
Daytime dozing			
Never/rarely	211,888 (80.6)	4,142 (77.3)	< 0.001
Sometimes	45,750 (17.4)	1,034 (19.3)	
All of the time/often	5,293 (2.0)	183 (3.4)	
High blood pressure			
No	207,166 (78.8)	4,197 (78.3)	0.410
Yes	55,765 (21.2)	1,162 (21.7)	
Diabetes			
No	253,331 (96.3)	5,142 (96.0)	0.134
Yes	9,600 (3.7)	217 (4.0)	
Anxiety/Depression			
Mean ± SD	2.99 ± 2.31	3.21 ± 2.43	< 0.001
SII			
Mean ± SD	586 ± 338	602 ± 338	< 0.001
NLR			
Mean ± SD	2.30 ± 1.16	2.30 ± 1.05	0.702
PLR			
Mean ± SD	142 ± 60.8	143 ± 56.1	0.233
LMR			
Mean ± SD	4.65 ± 3.94	4.80 ± 2.58	< 0.001

Normally distributed continuous variables displayed as the means (SDs), nonnormally distributed continuous variables displayed as medians (IQRs), and categorical variables displayed as numbers (percentages). SD, standard deviation; IQR, interquartile range; BMI, body mass index; LMR, lymphocyte-to-monocyte ratio; NLR, neutrophil-to-lymphocyte ratio; PLR, platelet-to-lymphocyte ratio; SII, systemic immune-inflammation index.

Compared with participants without incident IBS, those with incident IBS were more likely to be younger, female, to have less exercise, work more shifts, have shorter sleep duration, more likely to be an “evening person,” usually suffer from insomnia, do not snore, always be sleepy during the day, and had higher PHQ-4 and SII and LMR scores.

### Shiftwork and intestinal diseases

During a mean follow-up of 13.7 years, a total of 5,359 (1.997%) participants developed IBS. Shiftwork status was classified as always/usually, sometimes and never/rarely. Compared to participants with never/rarely rotating shift work, those adherence to always/usually rotating shift work had a significantly higher risk of developing IBS, (HR: 1.12, 95% CI: 1.03–1.23; Table 2), but no significant associations were found with other intestinal diseases (for Crohn, (HR: 1.20, 95% CI: 0.93–1.60); for intestinal vascular disease, (HR: 1.20, 95% CI: 0.96–1.40); for ulcerative colitis, (HR: 1.10, 95% CI: 0.94–1.30); and for colorectal cancer, (HR: 1.01, 95% CI: 0.89–1.15), respectively; Supplementary Table S3). Although for paralytic intestinal obstruction the association is significant, (HR: 1.20, 95% CI: 1.10–1.30), the statistical power is not sufficient to indicate an association between them.

**Table 2:** Association between job involves shiftwork and the incidence of IBS.

	Cases of IBS	No of non-IBS	Crude model			Model 1			Model 2		
			HR	Lower 95%CI	Upper 95%CI	HR	Lower 95%CI	Upper 95%CI	HR	Lower 95%CI	Upper 95%CI
Job involves shiftwork											
Never/rarely	4,381	217,328	ref			ref			ref		
Sometimes	390	19,582	0.99	0.89	1.10	1.05	0.95	1.17	1.02	0.92	1.14
Always/usually	588	26,021	1.12	1.03	1.23	1.19	1.09	1.29	1.12	1.03	1.23

Crude model was adjusted for: none. Model 1 was adjusted for: age, Townsend Deprivation Index, sex, ethnicity. Model 2 was further adjusted for: optimal physical activity, BMI, smoking status, tea intake, alcohol drinking, healthy diet score, chronotype preference, sleep duration, sleeplessness insomnia, snoring, daytime dozing, high blood pressure diagnosed by doctor, diabetes diagnosed by doctor, anxiety/depression, LMR, PLR, NLR, SII based on Model 1.

### Subgroup and sensitivity analyses

**Figure 2:**
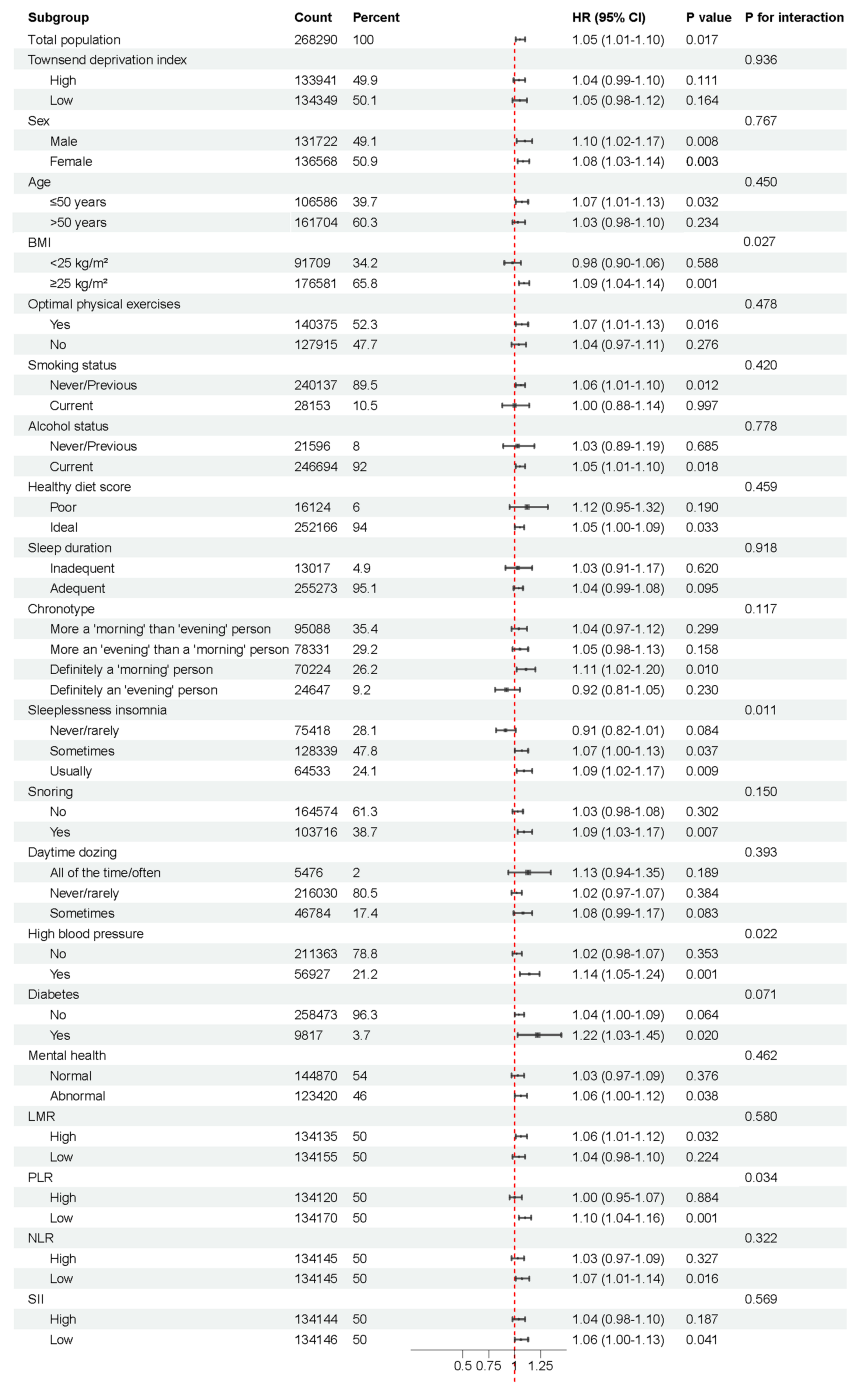
Subgroup analysis of shift work conditions and the risk of IBS. Possible modifications were assessed for variables including age, sex, ethnicity, Townsend deprivation index, BMI, smoking status, alcohol drinking, diabetes diagnosed by doctor, high blood pressure diagnosed by doctor, healthy diet score, optimal physical activity, sleep duration, chronotype, sleeplessness insomnia, snoring, daytime dozing, anxiety/depression, LMR, PLR, NLR, SII.

In subgroup analysis, we found significant association between rotating shift work and the risk of IBS among participants with sometimes or usually sleeplessness insomnia and both sexes (for never/rarely sleeplessness insomnia, (HR: 0.91, 95% CI: 0.82–1.01); for sometimes sleeplessness insomnia, (HR: 1.07, 95% CI: 1.00–1.13); for usually sleeplessness insomnia, (HR: 1.09, 95% CI: 1.02–1.17); for males, (HR: 1.10, 95% CI: 1.02–1.17); and for females, (HR: 1.08, 95% CI: 1.03–1.14), respectively), shiftwork interacted with sleeplessness insomnia on the risk of IBS (P for interaction = 0.011). Significant modification effects by high blood pressure diagnosed by doctor and level of PLR were also detected when using per SD change (P for interaction = 0.022 for High blood pressure diagnosed by doctor and P for interaction = 0.034 for level of PLR). Additionally, shiftwork interacted with BMI on the risk of IBS (P for interaction = 0.028), with the risks generally observed among participants whose BMI ≥ 25 kg/m^2^ but not BMI < 25 kg/m^2^ (Figure 2).

Moreover, the increased IBS risk (not significant) associated with shiftwork was generally observed across TDI, sex, age, BMI, optimal physical activity, smoking status, alcohol drinking, healthy diet score, sleep duration, snoring, daytime dozing, high blood pressure diagnosed by doctor, diabetes diagnosed by doctor, anxiety/depression, LMR, PLR, NLR, SII. The risk of IBS did not differ based on chronotype, except for definitely a ‘morning’ person subgroup (Figure 2).

In order to assess the robustness of our findings, we conducted several sensitivity analyses. We did not find significant changes of the impact of shift work on IBS risk after excluding subjects with follow-up time < 1 year from baseline (HR: 1.14, 95% CI: 1.04–1.24) or the model of the dataset excluding non-White participants (HR: 1.15, 95% CI: 1.05–1.26). The results showed no substantial change of the impact of shift work on IBS (Supplementary Tables S4, S5).

### Joint analysis of IBS

**Figure 3:**
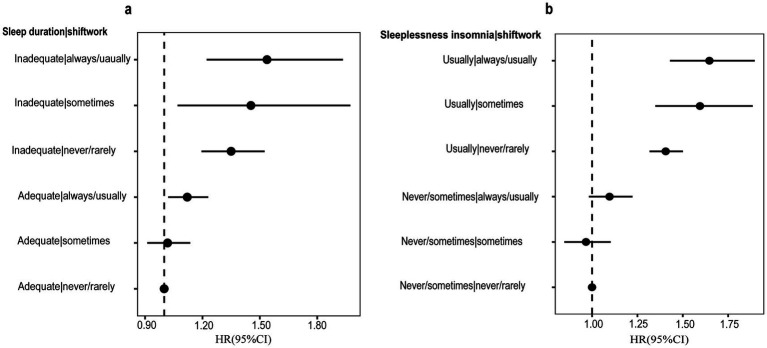
The joint association of **(a)** sleep duration **(b)** sleeplessness insomnia and shiftwork conditions with the incidence for IBS. Sleep duration were categorized into: adequate, < 6 h; inadequate, ≥ 6 h. Sleeplessness insomnia were categorized into: usually and never/sometimes, ≥ 6 h. Models were adjusted for age, Townsend Deprivation Index, sex, ethnicity, optimal physical activity, BMI, smoking status, tea intake, alcohol drinking, healthy diet score, chronotype preference, sleep duration, sleeplessness insomnia, snoring, daytime dozing, high blood pressure diagnosed by doctor, diabetes diagnosed by doctor, anxiety/depression, LMR, PLR, NLR, SII.

After including sleep duration and shift work interaction terms in the cox proportional hazard models, we found evidence of a joint association of sleep duration and rotating shift work with the incidence of IBS. Compared to those with both adequate sleep duration and never/rarely shift work, participants with both adequate sleep duration and sometimes shift work was positively associated with risk of IBS (HR: 1.02, 95% CI: 0.91–1.14), although the statistical significance is not significant. Participants with both adequate sleep duration and always/usually shift work had a 12% increased risk of IBS (HR: 1.12, 95% CI: 1.02–1.23), 35% increased risk (HR: 1.35, 95% CI: 1.19–1.52) for those with both inadequate sleep duration and never/rarely shift work, and 45% increased risk (HR: 1.45, 95% CI: 1.07–1.97) for those with both inadequate sleep duration and sometimes shift work. Of note, those with both inadequate sleep duration and always/usually shift work had a 54% increased risk of IBS (HR: 1.54, 95% CI: 1.35–1.82), which is the highest risk of all the combinations (Figure 3a).

Similarly, Figure 3b illustrated the HR for each combination of sleeplessness insomnia and rotating shift work interaction terms. Compared to those with never/sometimes insomnia and never/rarely shift work, participants with both never/sometimes insomnia and always/usually shift work was positively associated with risk of IBS (HR: 1.10, 95% CI: 0.98–1.22), although the statistical significance is not significant. Participants with both usually insomnia and never/rarely shift work had a 41% increased risk (HR: 1.41, 95% CI: 1.32–1.50), and 59% increased risk (HR: 1.59, 95% CI: 1.32–1.88) for those with both usually insomnia and sometimes shift work. It is worth noting that those with both usually insomnia and always/usually shift work had the highest increased risk of IBS, which is 65% (HR: 1.65, 95% CI: 1.43–1.90) higher than those with never/sometimes insomnia and never/rarely shift work (Figure 3b).

### Mediation analysis

We observed significant mediating effects by healthy sleep and anxiety/depression of the association between job involves shift work and IBS incidence (Table 3). The mediation analysis showed that the association between shift work and IBS was partially mediated by sleep health (Mediation proportion = 16.10%, *p* < 0.001) with pure natural indirect effects of 0.011 (0.009–0.014) and anxiety/depression (Mediation proportion = 3.60%, *p* = 0.029) with pure natural indirect effects of 0.002 (0.001–0.003).

**Table 3:** Estimated proportion of associations between job involves shiftwork and IBS mediated by sleep quality and anxiety/depression.

Mediator	Job involves shiftwork			
	Proportion mediated (95% CI)	P	Pure natural indirect effects	P
Sleep quality	0.161 (0.068,0.254)	< 0.001	0.011 (95% CI: 0.009–0.014)	< 0.001
Anxiety/Depression	0.036 (0.004, 0.068)	0.029	0.002 (95% CI: 0.001–0.003)	0.001

## Discussion

In this population-based prospective cohort study, we observed an increased risk of IBS among shift workers compared with non-shift workers. The harmful effect of rotating shift work was partially explained by anxiety/depression and sleep quality. Joint association indicated that inadequate sleep duration and usually insomnia can help intensify the deleterious associations of rotating shift work with the incidence of IBS.

The positive association between IBS and shift work in our study was consistent with several previous studies. A prior cross-sectional study included 207 nurses and nursing assistants in Korea revealed that the prevalence of IBS in rotating shift worker group was significantly higher than that of daytime worker group, while the sample size is too small and the population is limited (14). Another study at a tertiary Australian hospital also indicated that irregular working hours appeared to be associated with gastrointestinal symptoms and functional gastrointestinal disorders (including IBS), but the study was a qualitative survey and included only 274 healthcare professionals (28). Similarly, the results of a meta-analysis showed that shift work was the major influencing factor of medical staff suffering from IBS (29). However, most of the previous studies have been conducted among doctors or nurses in hospitals (4, 30), there is a lack of evidence based on the general population. In contrast, our study had a larger sample size and a more representative study population of the entire UK workforce.

The study focuses on the significant association between IBS and rotating shift work, while no such associations were observed with other common gastrointestinal diseases (crohn, intestinal vascular disease, ulcerative colitis and colorectal cancer). Although a statistical significance was observed between shift work and the incidence of paralytic intestinal obstruction, the sample size was too small to establish a conclusive link between rotating shift work and the disease. Therefore, the study highlights IBS as the primary focus, given the stronger statistical evidence of its connection to rotating shift work.

Several potential mechanisms might contribute to this increased risk of IBS in shiftwork participants. The gut-brain-microbiota axis plays a crucial role in the pathophysiology of IBS and the development of conditions linked to shift work and sleep quality. Shift work, particularly in rotating and night shifts, disrupts the body’s circadian rhythm and can significantly impact gut microbiota composition, leading to dysbiosis. This disruption influences the bidirectional communication between the gut and brain, aggravating IBS symptoms (31). For instance, the altered microbiota may contribute to gastrointestinal hypersensitivity, motility issues, and an immune response, which are hallmark features of IBS (10). Additionally, neurotransmitters such as serotonin (5-HT) play a pivotal role in gut-brain communication, as they affect intestinal motility, immune activation, and GI inflammation (12). The disruption of gut microbiota due to shift work can impact serotonin signaling, thus amplifying symptoms of IBS (32). Moreover, shift work-induced disturbances in sleep are closely linked to changes in gut microbiota composition, which further perpetuates neuropsychiatric conditions like anxiety and depression (33). The bidirectional communication between the gut and brain, facilitated by the microbiota, suggests that restoring gut balance through interventions like probiotics could help mitigate the effects of shift work on both IBS and mental health (13).

Subgroup analysis indicates that the association between shift work and IBS is especially pronounced in certain populations, possibly due to underlying factors that amplify vulnerability to IBS. High BMI is linked to a heightened risk of IBS, likely due to the inflammatory nature of obesity; obesity stimulates the release of proteins such as complement components and CRP, contributing to IBS symptoms (34). Insomnia also plays a role, by weakening the immune system as well as increasing the expression of proinflammatory cytokines, which enhances sensitivity to visceral pain, leading to an overreaction to normal gastrointestinal activity in patients with IBS and exacerbating symptoms (35). Hypertension is another factor associated with a higher risk of IBS, potentially due to shared pathological mechanisms, such as intestinal barrier dysfunction and systemic low-grade inflammation (36). Increased intestinal permeability allows bacterial endotoxins to enter the bloodstream triggering pro-inflammatory cytokine release, which not only worsens IBS symptoms but may also contribute to cardiovascular conditions, including hypertension (37).

### Strength

First, a large number of participants provided a detailed employment history, allowing us to categorize the frequency of shifts, thus overcoming limitations of many previous studies. Second, individuals in the UK Biobank were recruited entirely independent of employment status, ensuring that the sample was representative of the entire UK workforce. This approach effectively mitigated the potential selection bias that could arise from studying a single occupation. Third, large-scale prospective cohort studies have explored the joint effect of sleep and shift work, and found that there is a synergistic effect between them, which has enriched the research on related mechanisms. Meanwhile, combined with inflammatory markers, the potential mechanism of physiological circadian rhythm and IBS pathogenesis was explored. Furthermore, we were able to explore the potential mediation pathway by sleep health and anxiety/depression in this association.

### Limitation

Firstly, despite the substantial sample size, our analysis is confined to adults aged 40 to 69 years, as the UK Biobank does not provide data on younger individuals and is predominantly composed of a White population. Consequently, the findings may necessitate validation across other age groups and racial or ethnic backgrounds. Secondly, only the baseline shift schedules were counted, but in reality, participants’ work status might change over time during the follow-up while people tend to stop doing shift or night shift work at an older age, which might bias our results toward the null hypothesis, resulting in an underestimation of the effect size. Thirdly, IBS might be misdiagnosed or underdiagnosed, hence some IBS cases might not be captured by EHRs. Fourthly, the Rome criteria, which define disorders of gut-brain interaction, are extensively applied in epidemiologic research, pathophysiologic studies, treatment trials, and clinical practice (38). However, diagnoses in the UK biobank are coded using ICD-10, and we did not have access to information on diseases diagnosed using the ROME criteria. Thus, this is one of the limitations of our study.

## Conclusion

In summary, the study found that adhering to shift work significantly increases the risk of IBS. Additionally, our results support the value of interventions to target anxiety/depression and sleep quality to reduce the incidence of IBS. Future prospective studies with device-based sleep and anxiety/depression assessments and trials targeting both status of health is warranted.

## Data Availability

The datasets presented in this study can be found in online repositories. The names of the repository/repositories and accession number(s) can be found in the article/Supplementary material.

## Footnotes

^1^ https://www.r-project.org
