# Supplementary material for: Association of rotating shift work with incident irritable bowel syndrome: a large population-based prospective cohort study
Source: Front Public Health. 2025 Mar 26;13:1541122. doi: 10.3389/fpubh.2025.1541122 (PMC11978828; doi:10.3389/fpubh.2025.1541122)
Supplement: Supplementary file 1 [file Table_1.DOCX]

**Supporting Information Appendix**

**Supplementary Table S1**. ICD-10 codes and Field ID defining diseases in UKB.

**Supplementary Table S2**. Healthy sleep score calculated.

**Supplementary table S3.** Hazard ratios for other intestinal diseases associated with rotating shiftwork.

**Supplementary Table S4**. Hazard ratios for primary outcome associated with current work schedule after excluding participants with follow-up time < 1 year from baseline(n = 267840).

**Supplementary Table S5.** Hazard ratios for primary outcome associated with current work schedule in the data excluding non-White participants.

| **Supplementary table S1.** ICD-10 codes and Field ID defining diseases in UKB. | | |
| --- | --- | --- |
| **Disease** | **ICD-10** | **Data field ID in UKB** |
| Irritable bowel syndrome | K58 | 131638; 131639 |
| Crohn's disease | K50 | 131626; 131627 |
| Ulcerative Colitis | K51 | 131628; 131629 |
| Celiac disease | K90 | 131688; 131689 |

| **Supplementary table S2.** Healthy sleep score calculated. | | | | |
| --- | --- | --- | --- | --- |
| **Characteristics** | **UK Biobank code** | **UK Biobank questionnaire** | **Healthy answer** | **Unhealthy answer** |
| Chronotype | 1180 | Do you consider yourself to be? | Definitely a "morning" person; More a "morning" than "evening" person | More an "evening" than a "morning person, definitely an "evening" person |
| Sleep Duration | 1160 | About how many hours sleep do you get in every 24 hours? (please include naps) | 7-8 hr./d | < 7 or ≥ 9 hr./d |
| Insomnia | 1200 | Do you have trouble falling asleep at night or do you wake up in the middle of the night? | Never/rarely; Sometimes | Usually |
| Snoring | 1210 | Does your partner or a close relative or friend complain about your snoring? | No | Yes |
| Daytime dozing/sleeping | 1220 | How likely are you to doze off or fall asleep during the daytime when you don't mean to? (e.g. when working, reading or driving) | Never/rarely; Sometimes | Often; All the time |

**Supplementary table S3.** Hazard ratios for other intestinal diseases associated with rotating shiftwork.

|  | **HR(95%CI)** | **P_Value** |
| --- | --- | --- |
| **Crohn** |  |  |
| JobinvolvesshiftworkAlways/Usually | 1.20 (0.93-1.60) | 0.15 |
| JobinvolvesshiftworkSometimes | 1.20 (0.89-1.60) | 0.23 |
| **Intestinal vascular disease** |  |  |
| JobinvolvesshiftworkAlways/Usually | 1.20 (0.96-1.40) | 0.12 |
| JobinvolvesshiftworkSometimes | 1.00 (0.79-1.30) | 0.98 |
| **Paralytic intestinal obstruction** |  |  |
| JobinvolvesshiftworkAlways/Usually | 1.20 (1.10-1.30) | 0.00 |
| JobinvolvesshiftworkSometimes | 1.20 (1.00-1.30) | 0.01 |
| **Ulcerative colitis** |  |  |
| JobinvolvesshiftworkAlways/Usually | 1.10 (0.94-1.30) | 0.19 |
| JobinvolvesshiftworkSometimes | 1.00 (0.84-1.30) | 0.76 |
| **Colorectal cancer** |  |  |
| JobinvolvesshiftworkAlways/Usually | 1.01 (0.89-1.15) | 0.85 |
| JobinvolvesshiftworkSometimes | 1.08 (0.94-1.24) | 0.30 |

Model was adjusted for: Age, Sex.

| **Supplementary table S4.** Hazard ratios for primary outcome associated with current work schedule after excluding participants with follow-up time < 1 year from baseline(n=267840) | | | | | | | | | | |  |
| --- | --- | --- | --- | --- | --- | --- | --- | --- | --- | --- | --- |
|  | **Total** | **Crude model** | | | **Model 1** | | | **Model 2** | | |  |
|  |  |  |  |  |  |  |  |  |  |  |  |
|  |  | **HR** | **Lower 95%CI** | **Upper 95%CI** | **HR** | **lower 95%CI** | **Upper 95%CI** | **HR** | **lower 95%CI** | **Upper 95%CI** |  |
| **Job involves shiftwork** |  |  |  |  |  |  |  |  |  |  |  |
| Never/rarely | 221329 | ref |  |  | ref |  |  | ref |  |  |  |
| Sometimes | 19946 | 1.02 | 0.91 | 1.13 | 1.08 | 0.97 | 1.21 | 1.05 | 0.95 | 1.17 |  |
|  |  |  |  |  |  |  |  |  |  |  |  |
| Always/Usually | 26565 | 1.13 | 1.04 | 1.24 | 1.2 | 1.1 | 1.31 | 1.14 | 1.04 | 1.24 |  |
|  |  |  |  |  |  |  |  |  |  |  |  |
| Crude model was adjusted for: None.  Model 1 was adjusted for: Age, Townsend Deprivation Index, Sex, Ethnicity.  Model 2 was further adjusted for: Optimal physical activity, BMI, Smoking status, Tea intake, Alcohol drinking status, Healthy diet score, Chronotype, Sleep Duration, Sleeplessness insomnia, Snoring, Daytime dozing, High blood pressure diagnosed by doctor, Diabetes diagnosed by doctor, anxiety/depression, LMR, PLR, NLR, SII based on Model 1. | | | | | | | | | | |  |

**Supplementary table S5.** Hazard ratios for primary outcome associated with current work schedule in the data excluding non-White participants.

|  | **Total** | | **Crude model** | | | **Model 1** | | | **Model 2** | | | |  |
| --- | --- | --- | --- | --- | --- | --- | --- | --- | --- | --- | --- | --- | --- |
|  |  |  |  |  |  |  |  |  |  |  |  |  |  |
|  |  |  | **HR** | **Lower 95%CI** | **Upper 95%CI** | **HR** | **lower 95%CI** | **Upper 95%CI** | **HR** | **lower 95%CI** | **Upper 95%CI** |  | |
|  |  |  |  |  |  |  |  |  |  |  |  |  | |
| Job involves shiftwork | |  |  |  |  |  |  |  |  |  |  |  | |
| Never/rarely | 221329 | | ref |  |  | ref |  |  | ref |  |  |  | |
| Sometimes | 19946 | | 1.02 | 0.92 | 1.14 | 1.08 | 0.97 | 1.20 | 1.04 | 0.94 | 1.16 |  | |
| Always/Usually | 26565 | | 1.16 | 1.06 | 1.27 | 1.22 | 1.12 | 1.34 | 1.15 | 1.05 | 1.26 |  | |
|  |  |  |  |  |  |  |  |  |  |  |  |  | |
| Crude model was adjusted for: None.  Model 1 was adjusted for: Age, Townsend Deprivation Index, Sex.  Model 2 was further adjusted for: Optimal physical activity, BMI, Smoking status, Tea intake, Alcohol drinking status, Healthy diet score, Chronotype, Sleep duration, Sleeplessness insomnia, Snoring, Daytime dozing, High blood pressure diagnosed by doctor, Diabetes diagnosed by doctor, Anxiety/Depression, LMR, PLR, NLR, SII based on Model 1. | | | | | | | | | | | | |  |
